# Combination of inverted pyramidal nanovoid with silver nanoparticles to obtain further enhancement and its detection for ricin

**DOI:** 10.1186/s11671-015-0806-6

**Published:** 2015-02-28

**Authors:** Meng Wang, Bin Wang, Shixuan Wu, Tingke Guo, Haoyu Li, Zhaoqing Guo, Junhua Wu, Peiyuan Jia, Yuxia Wang, Xiaoxuan Xu, Yufang Wang, Cunzhou Zhang

**Affiliations:** The MOE Key Laboratory of Weak Light Nonlinear Photonics, School of physics and Teda Applied Physics Institute, Nankai University, Tianjin, 300071 China; Institute of Pharmacology and Toxicology, Academy of Military Medical Sciences, Beijing, 100850 China

**Keywords:** Surface-enhanced Raman scattering, Ricin toxin, Combined substrate, Finite element method, 73.20.Mf, 74.25.nd, 81.07.-b

## Abstract

We have obtained the surface-enhanced Raman scattering substrate by depositing silver nanoparticles on the surface of the inverted pyramidal nanovoid in order to improve the enhance effects. Experimental results showed that the combined substrate exhibited greater enhancement than the nanovoid substrate or nanoparticles. In order to test the SERS activity of the combined substrates, Rh6G and ricin toxin were used as Raman probes. Finite element method was employed to simulate electric field and induced charge distribution of the substrates, which have been used to explore the interaction between nanoparticles and nanovoid as well as mechanism of the great enhancement.

## Background

As the discovery of surface-enhanced Raman scattering (SERS) phenomenon [1,2], researchers have been working on the enhance mechanism, improvement, and practical applications. During the past several decades, it has been a fruitful research area and promoted Raman spectra to join the ranks of single molecule detection [3,4]. Nowadays, the huge enhanced factor is generally considered to be the result of two kinds of enhancements: one is the electromagnetic contribution (EM enhancement) which arises from the surface plasmon oscillation; the other is the chemical enhancement which involves chemical bonding and charge transfer. Moreover, the EM enhancement is considered to play a major role [5,6].

Based on the synthesis methods, there are two kinds of SERS substrates, nanostructures and nanoparticles (NPs), both can localize the optical energy into nanoscale and get field enhancement. To benefit from the nano-fabrication techniques, various metallic plasmonic nanostructures have been manufactured and used as the enhance substrates, such as bowtie and X-shaped nanostructures [7,8], truncated spherical nanocavities [9], Klarite substrate [10], sub-10-nm gap structure [11] nano-mushroom and nano-ring arrays [12,13], and so forth. Noble metal nanoparticles of different shapes, dimensions, and compositions including the typical spherical, cubic [14,15], prismatic nanoparticles [16], ‘sea-urchin’-like particles [17], spiky nanoshells [18], and Ag-Fe_3_O_4_ nanocomposites [19] have been synthesized and utilized in SERS. Here, we demonstrate that the further enhancement, which is stronger than solitary nanostructure or nanoparticles, can be obtained by combining nanostructures with nanoparticles.

With the advancement of SERS technique, researchers are not satisfied with the detection of routine analytes such as pyridine, rhodamine, and benzene thiol; instead, they utilize SERS in more wide applications for sensing molecules in trace amounts within the field of chemical and biochemical analytics, such as detecting DNA molecule [20], bacteria [21], cocaine, heroin [22,23], and explosives [24]. We have detected and distinguished a kind of phytotoxin, ricin, using our combined SERS substrate. Compared with other detection method, such as enzyme-linked immunosorbent assay (ELISA) [25], SERS can identify the toxin rapidly with only a small amount of sample.

## Methods

### Substrate fabrication

Silver nitrate (AgNO_3_, 99 + %), poly(vinyl pyrrolidone) (PVP), and ethanol were purchased from Tianjin Jiangtian Chemical. Co. and used as received without further purification. Deionized water was prepared by a Milli-Q academic H_2_O purification system and used throughout the experiment.

**Figure 1:**
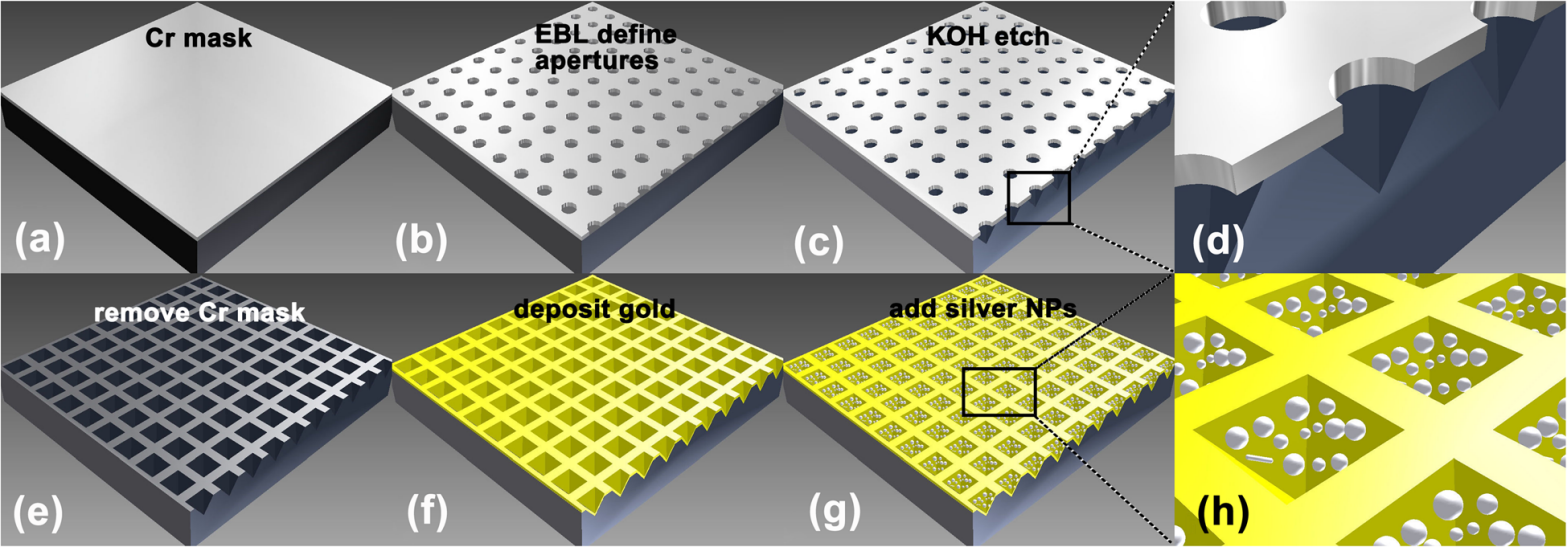
**Nanostructure substrate fabrication process. (a)** Deposit a layer of Cr on (100) oriented silicon wafer. **(b)** Define arrays of apertures on Cr mask by EBL. **(c)** Anisotropic KOH etching. **(e)** Remove Cr mask. **(f)** Deposit 200nm thick gold film. **(g)** Drop silver colloid onto the substrate. **d** and **h** are zoomed from **c** and **g**, respectively.

**Figure 2:**
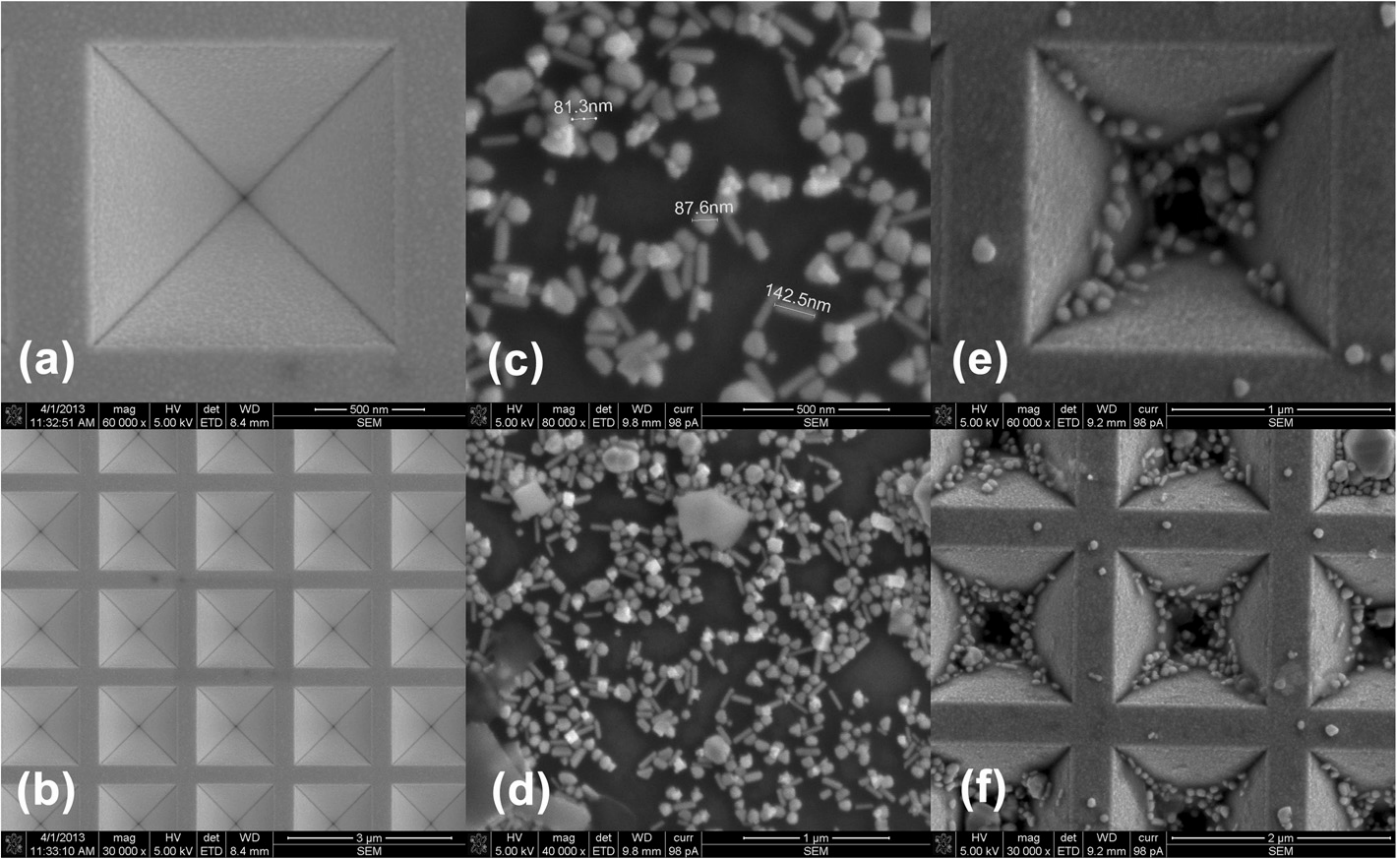
**SEM images of prepared samples. a** and **b** are SEM images of the inverted pyramidal nanovoid substrate. The pithead boundary length of the nanovoid is about 1.41 μm, its depth 1 μm, and its period 2 μm. **c** and **d** are SEM images of the prepared silver nanoparticles by solvothermal method. **e** and **f** are SEM images of the combined substrate.

The nanostructure substrates used in this study were fabricated from (100) oriented silicon wafers. As shown in Figure 1b,c,e, after defining arrays of apertures by electron-beam lithography (EBL) on Cr mask, anisotropic KOH etching to the {111} planes and removing the mask, the inverted pyramidal nanovoid arrays were formed (Figure 1e). Apex angle of the nanovoid was fixed at 70.5° [26]. By adjusting etching time, the depth of the nanovoid can be controlled. A layer of gold with thickness of 200 nm was sputtered onto it. Scanning electron microscope (SEM) images of the inverted pyramidal nanovoid substrate are shown in Figure 2a,b.

The silver nanoparticles were prepared by solvothermal method [27]. First, 0.017 g silver nitrate was dissolved in 10 ml ethanol. After stirring it completely, the solution was injected drop by drop using a syringe into 20 ml ethanol which contains 0.3 g PVP (K-30). The mixed solution was transferred into Teflon-lined stainless autoclaves and kept at 180°C in drying oven for 18 h. Then, it was cooled naturally to room temperature and centrifuged. The supernatant was removed, and the precipitate was dispersed in deionized water and centrifuged again. After repeating the process three times, the silver colloid was obtained.

As demonstrated by the SEM images in Figure 2c,d, the prepared silver colloid mainly consists of nanospheres and nanorods. The diameter of nanosphere is about 80 nm, and the length of nanorod is about 140 nm.

The combined substrate of gold-inverted pyramidal nanovoids and silver nanoparticles was prepared by electrostatic assembly method, in which the silver colloid was dropped onto the gold substrate and dried naturally. The SEM images of the combined substrate in Figure 2e,f show that most silver nanoparticles distribute in the voids, especially around the slope edge. Compared with that on the silicon wafer, the distribution of Ag NPs is denser.

### SERS experimental details

Rhodamine 6G (Rh6G) was used to characterize the SERS performance of the gold nanovoid, silver colloid, and combined substrate. 10^−5^ M Rh6G solution was chosen so that SERS signal could be obtained for all the three substrates under the same experimental condition. Rh6G solution was dropped onto the gold nanovoid or combined substrate. For the silver colloid enhancement, equivalent volume of colloid and Rh6G solution was mixed and dropped onto the surface of a polished silicon wafer. Raman spectra were collected by Renishaw confocal microscope Raman spectroscopy with 785-nm wavelength laser as an excitation source. The laser was focused on the substrates by a long working distance objective (×50 and NA = 0.5). The diameter of the focused laser beam is about 2 μm, and the power is 0.15 mW. The accumulation time is 10 s. For each enhanced substrates, six different points were randomly chosen to collect SERS spectra.

### Theoretical simulation details

Finite element method (FEM) [28] was employed to simulate electric field and induced charge distribution of gold nanovoid substrate, silver nanoparticle dimer, and combined substrate. Linearly polarized plane wave was used as the incident source. Perfect match layers (PMLs) were applied in propagation direction to avoid nonphysical reflection from boundaries. Periodic boundary conditions in *x* and *y* direction were used when simulating nanovoid substrate and combined substrate. The permittivity of gold and silver was referred to the experimental data of Johnson and Christy [29] and interpolated to get the dispersion relation.

## Results and discussion

### SERS spectra of Rh6G and ricin toxin

**Figure 3:**
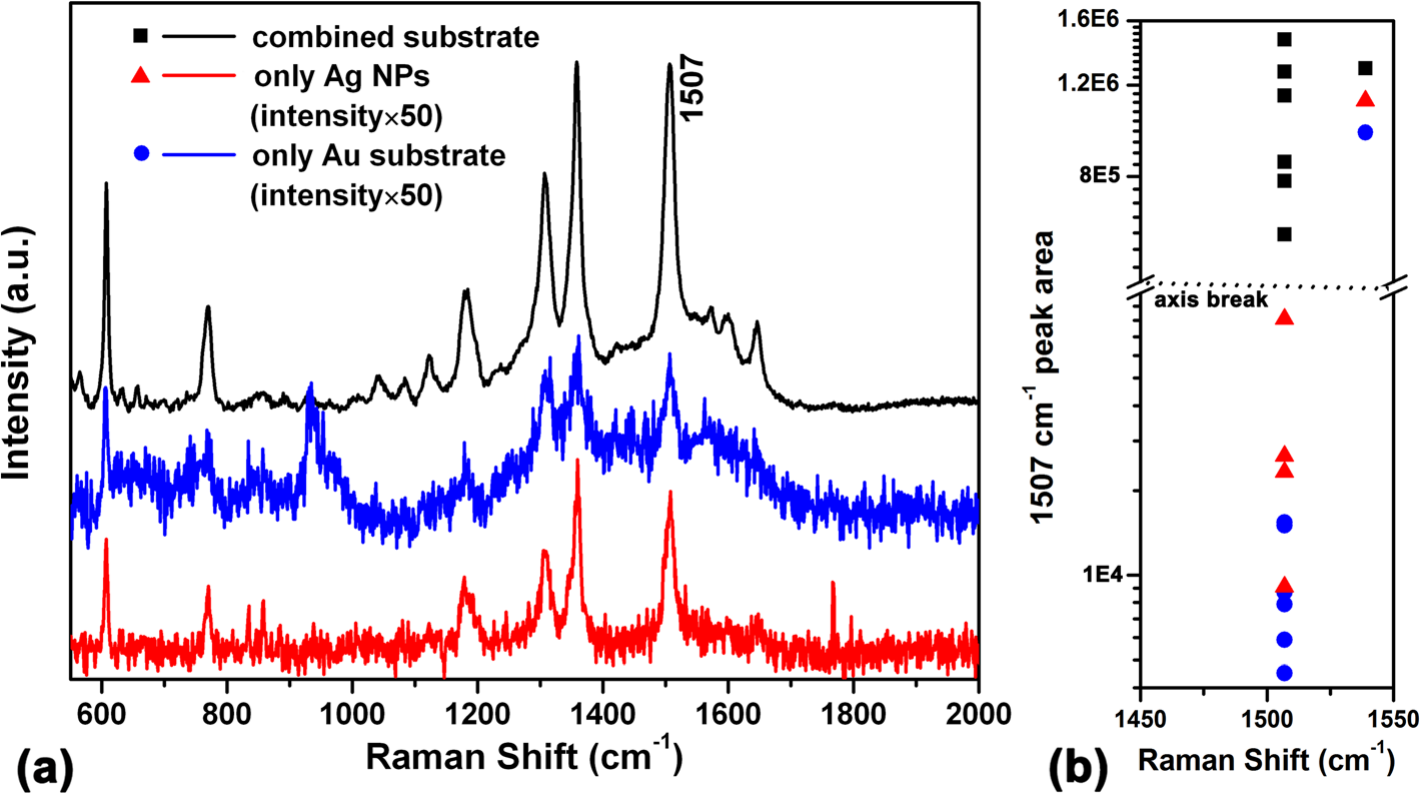
**Comparison of SERS spectra of Rh6G. (a)** Comparison of SERS spectra obtained by solitary gold nanovoid substrate, silver nanoparticles, and combined substrate. **(b)** 1,507 cm^−1^ peak area comparison. For visualization, a factor of 50 is multiplied to the spectra which correspond to gold substrate and silver nanoparticles in a, and the scale of vertical axis in b is taken logarithm.

A comparison of SERS spectra of Rh6G obtained from three types of substrates is illustrated in Figure 3a. It can be seen that the combined substrate demonstrates significant further enhancement. The scattering peak at 1,507 cm^−1^ is selected to compare the enhance performance. The peak area calculated from each spectrum is shown in Figure 3b. The average peak area obtained by combined substrate is about 44 times larger than that obtained by the solitary silver colloid and 107 times than that of the gold substrate.

In order to further investigate the enhancement effects of the combined substrate, SERS spectra of ricin with the concentration of 10 μg/ml in phosphate-buffer saline (PBS) were measured. Ricin is a kind of toxin which can be easily extracted from castor seeds. Purified ricin with the size of a few grains of table salt could be fatal for an adult human. Ricin protein consists of two chains of polypeptide, A chain (RTA) bound to B chain (RTB) by a single disulfide bond. RTA can remove an adenine from ribosomal RNA (rRNA), prevent synthesis of protein, and cause the death of cells. RTB is a lectin that binds to glycoproteins or glycolipids on the surface of target cells and helps RTA penetrating into the cell [30].

**Figure 4:**
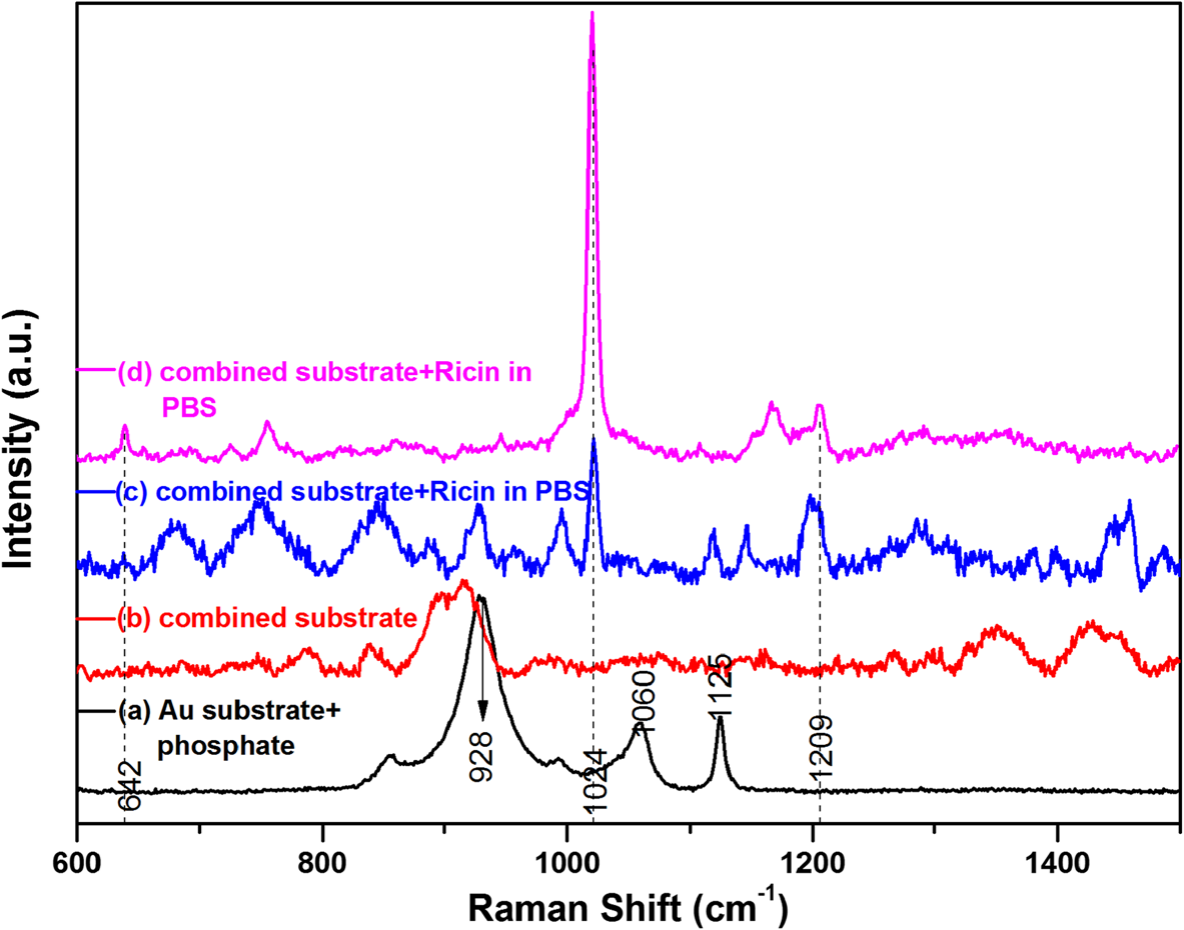
**Distinguishing Raman signal of ricin toxin.** Raman spectra collected from **(a)** phosphate on gold substrate, **(b)** combined substrate, and **(c,d)** ricin toxin in PBS on combined substrate.

In order to distinguish the Raman-scattered peaks of ricin from those of phosphate, Raman spectrum of phosphate solution was measured by dropping the solution onto the surface of the gold substrate. As shown in Figure 4a, the scattered peaks that appeared at 928, 1,060, and 1,125 cm^−1^ belong to phosphate, in agreement with the other report [31]. Another type of silver colloid, in which ethylene glycol instead of ethanol was chosen to reduce silver nitrate [32], was prepared to combine with the gold substrate. Raman spectra for naked combined substrate and ricin on the combined substrate were collected, as illustrated in Figure 4b-d. From Figure 4c, it can be seen that a new scattered peak which is distinguished from those of phosphate and the naked substrate appeared at 1,024 cm^−1^. As can be seen from Figure 4d, a much stronger scattering peak at 1,024 cm^−1^ was obtained. Besides this major peak, other scattered peaks that appeared at 642 cm^−1^ and 1,209 cm^−1^ were also observed. These Raman bands are likely attributed to protein of ricin toxin.

### Theoretical simulation results

**Figure 5:**
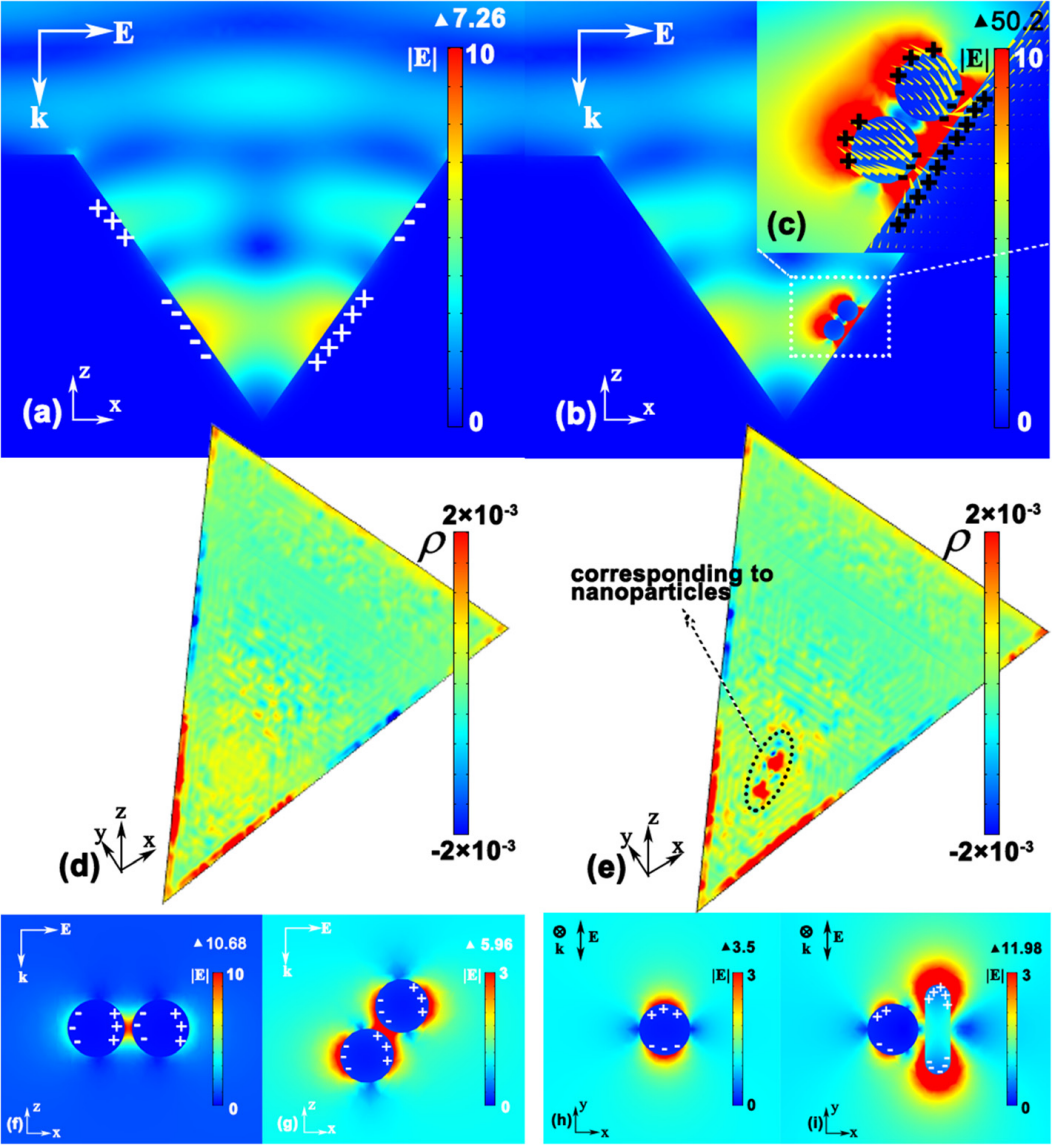
**FEM-simulated field and charge distribution.** Near-field electric intensity and induced charge distribution of **(a)** the periodic inverted pyramidal nanovoid, **(b)** combined nanostructure, **(f)** horizontally placed silver nanoparticle dimer, **(g)** obliquely placed dimer, **(h)** single silver nanosphere, **(i)** silver nanosphere, and nanorod. The tilt angles of silver dimer in b and g are the same. **c** is enlarged from b. **d** and **e** display induced charge density distribution on the surface of the nanovoid right side wall, corresponding to nanovoids shown in a and b, respectively.

In order to explore the mechanism of the further enhancement property of the combined substrate, finite element method was employed to simulate the near-field electric field distribution of the substrate under light illumination. E-field distributions of solitary gold nanovoid substrate and silver nanoparticle dimer were also calculated for comparison. As shown in Figure 5a,b,f, the plane wave which polarizes along the *x*-axis is incident from the top. The wavelength is 785 nm, which is the typical laser wavelength of Raman spectroscopy. The diameters of silver nanospheres in Figure 5b,f-i are 80 nm. The pithead boundary length of pyramidal nanovoid in Figure 5a,b is set to be 1.41 μm, depth 1 μm, and period 2 μm, in accordance with the size of the experimental substrate as shown in Figure 2. In Figure 5, all the gaps between nanoparticles or nanoparticle and nanovoid surface are 10 nm. The maximum of the color bar in Figure 5a,b,f is set to 10 V/m, which is convenient for comparison.

It can be easily observed from Figure 5b that there is a much stronger and larger area electric field distribution around the silver nanoparticles when combined with gold nanovoid. In this case, the maximum value of electric field intensity can be five times larger than that of the solitary gold nanovoid substrate or silver nanoparticle dimer. The large near-field enhancement can be explained by the double interaction between the combined substrate and incident light. First, the incident light is coupled into inverted pyramidal nanovoid. Then, the concentrated near fields in nanovoid have been further enhanced by silver nanoparticles, which result in a large near-field enhancement of combined substrate.

Schematic diagram of induced charge distribution is also plotted in Figure 5, from which we can observe that charge distribution is affected by the polarization of incident e-field and the interaction between metal nanoparticles. It can be observed from Figure 5g that the charge distribution of silver nanosphere has deviated from the polarization direction of incident e-field, due to the interaction of induced charges between two metal spheres. In the case of Figure 5b, because of the existence of nanovoid side wall, interaction between nanoparticle and the side wall becomes dominant. By comparing induced charge distribution on the surface of nanovoid right side wall with (Figure 5e) or without (Figure 5d) the silver nanoparticle dimer, it can be seen that nanoparticles play a role of gathering charges. It is attributed to the appealing effect between charges on the surface of nanoparticles and nanovoid side wall. This effect causes accumulation of different charges on two sides of the gap, which promotes the oscillation of electron gas and its coupling with localized field, resulting in giant field enhancement.

In addition to the appealing effect of induced charges, there also exist the repelling effect due to the nanoparticle interaction, which is illustrated in Figure 5h,i. In Figure 5h, the induced charge distribution of a single silver nanosphere (diameter 80 nm) is consistent with polarization of incident e-field. When another metal nanoparticle (such as a silver nanorod, length 140 nm, diameter 40 nm) is placed very near to it, the ‘hot areas’ migrate further from nanorod obviously, which is due to the repelling effect of the same charges between nanosphere and nanorod.

## Conclusions

We combined periodic gold nanovoid substrate with silver nanoparticles and obtained greater enhancement than solitary gold substrate or silver nanoparticles. SERS spectra were measured to characterize its enhancing property. Besides Rh6G, ricin toxin was also detected using the combined substrate. Theoretical simulation showed that much stronger and larger area electric field distribution, which was attributed to double interaction between the combined substrate and incident light, appeared around silver nanoparticles when combined with gold nanovoid. Induced charge distribution revealed the appealing or repelling effect between nanostructures.

## Abbreviations

SERS: surface-enhanced Raman scattering
EM: electromagnetic
NPs: nanoparticles
ELISA: enzyme-linked immunosorbent assay
PVP: poly(vinyl pyrrolidone)
EBL: electron-beam lithography
SEM: scanning electron microscope
Rh6G: rhodamine 6G
FEM: finite element method
PML: perfect match layer
PBS: phosphate-buffer saline
RTA: ricin A chain
RTB: ricin B chain
